# Comparative Proteomic Analysis of *Streptococcus suis* Biofilms and Planktonic Cells That Identified Biofilm Infection-Related Immunogenic Proteins

**DOI:** 10.1371/journal.pone.0033371

**Published:** 2012-04-13

**Authors:** Yang Wang, Li Yi, Zongfu Wu, Jing Shao, Guangjin Liu, Hongjie Fan, Wei Zhang, Chengping Lu

**Affiliations:** 1 Key Lab of Animal Bacteriology, Ministry of Agriculture, Nanjing Agricultural University, Nanjing, China; 2 College of Animal Science and Technology, Henan University of Science and Technology, Luoyang, China; University of South Dakota, United States of America

## Abstract

*Streptococcus suis* (SS) is a zoonotic pathogen that causes severe disease symptoms in pigs and humans. Biofilms of SS bind to extracellular matrix proteins in both endothelial and epithelial cells and cause persistent infections. In this study, the differences in the protein expression profiles of SS grown either as planktonic cells or biofilms were identified using comparative proteomic analysis. The results revealed the existence of 13 proteins of varying amounts, among which six were upregulated and seven were downregulated in the *Streptococcus* biofilm compared with the planktonic controls. The convalescent serum from mini-pig, challenged with SS, was applied in a Western blot assay to visualize all proteins from the biofilm that were grown *in vitro* and separated by two-dimensional gel electrophoresis. A total of 10 immunoreactive protein spots corresponding to nine unique proteins were identified by MALDI-TOF/TOF-MS. Of these nine proteins, five (Manganese-dependent superoxide dismutase, UDP-N-acetylglucosamine 1-carboxyvinyltransferase, ornithine carbamoyltransferase, phosphoglycerate kinase, Hypothetical protein SSU05_0403) had no previously reported immunogenic properties in SS to our knowledge. The remaining four immunogenic proteins (glyceraldehyde-3-phosphate dehydrogenase, hemolysin, pyruvate dehydrogenase and DnaK) were identified under both planktonic and biofilm growth conditions. In conclusion, the protein expression pattern of SS, grown as biofilm, was different from the SS grown as planktonic cells. These five immunogenic proteins that were specific to SS biofilm cells may potentially be targeted as vaccine candidates to protect against SS biofilm infections. The four proteins common to both biofilm and planktonic cells can be targeted as vaccine candidates to protect against both biofilm and acute infections.

## Introduction

*Streptococcus suis* (SS) is a major worldwide pathogen and colonizes the respiratory tract of pigs, particularly the tonsils and nasal cavities [1]. SS is believed to be a normal inhabitant of several ruminants [2]. SS binds to the extracellular matrix (ECM) proteins, including fibronectin and collagen [3], of endothelial and epithelial cells [4], [5]. Some studies have demonstrated that SS has the ability to form biofilms [6], [7]. The biofilm mode of growth affords SS several advantages over its planktonic counterparts, including the capability of ECM to trap nutrients and protect against both antimicrobial agents and the host immune responses [6], [7]. Our previous studies indicate that SS maybe achieve persistent infections *in vivo* by forming biofilms [8] and hence SS infections might be difficult to treat. Biofilms play a key role in the pathogenesis and persistence of several bacterial infections [9]. It has been postulated that an altered metabolism and changes in gene expressions and protein amounts in biofilms may be responsible for drug resistance, cell adherence and virulence. Recent results indicate that biofilm cells have an active, although altered cell metabolism [10], [11]. Considerable investigation is required to gain a better understanding of biofilm formation.

Previous studies have investigated different immunogenic components of planktonically grown SS proteins; e.g., secreted or cell wall associated proteins using immunoproteomic assays [12], [13], [14], [15]. Zhang *et al.* reported that 11 membrane-associated proteins and nine extracellular proteins are immunogenic proteins using the hyperimmune or convalescent serum of minipigs [12], [13]. Geng *et al.* identified 32 proteins with high immunogenicity of which 22 were not previously reported [14]. Zhang *et al.* identified a total of 34 proteins by immunoproteomic analysis, of which 15 were recognized by both hyperimmune sera and convalescent sera [15]. At present, little is known about proteins targeted by the host immune system in the case of biofilm-mediated infections. Identifying those SS proteins that are targeted by the host immune system would increase the understanding of host defense mechanisms and help to identify novel means of diagnosis and treatment for pigs with persistent infections. Identification of these immunogenic antigens is necessary for effective vaccine design and to understand the molecular mechanisms that control biofilm formation by SS.

In this study, the differences in the whole cell protein expressions of SS cultivated under biofilm versus planktonic conditions were investigated. We utilized a convalescent mini-pigs model of challenged SS and an *in vitro* biofilm growth system to identify the immunogenic antigens of SS biofilm infections. We identified several proteins unique to SS grown as biofilms and planktonic cells by employing two-dimensional gel electrophoresis (2DGE) and matrix-assisted laser desorption ionization–time of flight–time of flight mass spectrometry (MALDI-TOF/TOF-MS) analysis.

## Materials and Methods

### Bacteria and Culture Conditions

SS2 strain clinical isolate HA9801 was used in this study. This strain was isolated by our laboratory in Jiangsu, China in 1998 and has the ability to form biofilms [8]. For biofilm cultures, SS was grown in THB medium (Oxoid, Wesel, Germany) supplemented with 1% fibrinogen in 100 mm polystyrene petri dishes at 37°C for 24 h. The supernatant was then removed and the plates were rinsed twice with 50 mM Tris/HC1 (pH 7.5). Biofilms were detached by scraping. Cells were sonicated for 5 min (Bransonic 220; Branson Consolidated Ultrasonic Pvt Ltd, Australia), followed by centrifugation at 12,000 × g for 10 min at 4°C and the supernatant was discarded. Cell pellets were washed twice with 50 mM Tris-HC1 (pH 7.5) by resuspending pellets with vortexing and collected by centrifugation 12,000 × g for 10 min at 4°C. SS planktonic cell was grown in 500 Erlenmeyer flasks containing 100 ml of the above culture medium at 37°C for 24 h. Planktonic cells were pelleted and washed as described above.

### Extraction of Proteins from SS Cells

Protein was extracted from SS cells as described by Rathsam [16] with minor modifications. Briefly, the SS cell pellets from biofilm and planktonic cultures were resuspended in buffer (Tris-HCl, MgCl_2_, 50% sucrose) supplemented with 1000 U/ml Mutanolysin (Sigma) and were incubated for 90 min at 37°C. The spheroplasts were collected and resuspended by sonication on ice at 100W for 90 cycles of (5 s on, 10 s off) using a sonication buffer (7 M urea, 2 M thiourea, 4% CHAPS, and 65 mM DTT), and incubated at 25°C for 30 min. The cell debris and unbroken cells were removed by centrifugation at 10,000 × g for 30 min at 25°C. The proteins in the supernatant were precipitated using 10% TCA at 4°C for 30 min. Precipitated protein was collected by centrifugation at 10,000 × *g* for 10 min at 4°C and washed twice with chilled acetone. The final pellet was air-dried. The dried pellet was dissolved in sample preparation solution, then incubated for 30 min at 25°C (vortexed every 10 min) and centrifuged at 10,000 ×g for 20 min at 25°C. Before rehydration, the supernatant was treated with a 2-D Clean-up Kit (GE Healthcare) to remove contaminants that can interfere with electrophoresis. The protein content was determined using the PlusOne 2-D Quant Kit (GE Healthcare) following manufacturer’s directions.

### 2-D Gel Electrophoresis

2DGE was performed using the immobiline/polyacrylamide system. Isoelectric focusing (IEF) was performed with IPG Drystrips (IPGphor; 13 cm; GE Healthcare) with 200µg of the protein sample using the in-gel sample rehydration technique according to the manufacturer’s instructions. IEF was performed in a Protein IEF Cell (GE Healthcare) using a stepwise voltage gradient to 80 kVh. Before the second dimension, strips were equilibrated for 2×15 min in equilibration buffer (6 M urea, 2% SDS, 30% glycerol, 0.05 M Tris–HCl pH 8.8), containing 1% DTT and 4% iodoacetamide, respectively. SDS-PAGE was carried out vertically in an Ettan DALT II system (GE Healthcare) using 12.5% polyacrylamide gels. Resolved proteins were stained with Coomassie Brilliant Blue G-250 stain for identifying the protein bands. All experiments were performed in triplicate. Reproducibility of the 2DGE was verified by analyzing the same samples at least three times on independent gels. Three replicate gels from three independent experiments were analyzed for each growth condition. The gels were analyzed using the Image Master Platinum 5.0 software (GE Healthcare). The normalized protein amount for each protein spot was calculated as the ratio of that spot volume to the total spot’s volume on the gel. Either Student t-test (P < 0.05) or a threshold of 2-fold change was used to determine significant difference between the two groups.

### Preparation of Convalescent Sera

Five specific pathogen free mini-pigs were injected with SS (1.0×10^8^ CFU/mL, 1 mL/pig, intramuscularly). As a control, preimmune sera were collected from mini-pigs before SS injection. Twenty days after the first injection, the survivor was again injected with second (identical) dose of SS. Serum was collected seven days after the second injection. The OD of the serum from pig injected with SS2 was 0.93±0.15 and the OD of the preimmune sera was 0.26 ± 0.05. The titers of the convalescent sera were evaluated by ELISA (unpublished protocol), and the sera with high titer was selected for subsequent experiments. All animal experimental protocols were approved by Science and Technology Agency of Jiangsu Province (SYXK-SU-2010-0005).

### Western Blotting

Protein samples from the 2DGE were transferred onto a PVDF membrane (GE Healthcare) using a semi-dry blotting apparatus (TE77, GE Healthcare) for 2 h at 0.65 mA/cm^2^. After transfer, the membrane was blocked with 100 mM Tris, 150 mM NaCl, 0.05% Tween-20 (TBST), containing 5% dry milk powder for 2 h. The blocked-membrane was then incubated with sera from either preimmune or convalescent mini-pigs (1∶1000 dilution) for 2 h at room temperature with gentle agitation. The membrane was washed three times with TBST buffer for 10 min per wash and incubated with horseradish peroxidase-labeled Staphylococcal protein A (Boster, Nanjing, China), (1∶5000 dilution) in blocking buffer for 1 h with gentle agitation. The membrane was washed as described above. The membranes were incubated with DAB substrate (Tiangen, Nanjing, China) for 10 min. Each sample was analyzed three times by western blot.

### Mass Spectrometry Analysis of Protein Spots and Database Searches

Differential expression spots and immune-reactive proteins were excised from the 2-D gels and sent to the Shanghai Applied Protein Technology Co. Ltd for trypsin in-gel digestion and MALDI-TOF-MS analysis. Protein spots with a low Mascot score were further analyzed using MALDI-TOF/TOF-MS to confirm identity. Data from MALDI-TOF-MS and MALDI-TOF/TOF-MS analysis were used in a combined search against the NCBInr protein database using MASCOT (Matrix Science) with the parameter settings of trypsin digestion, one max missed cleavages, variable modification of oxidation (M), and peptide mass tolerance for monoisotopic data of 100 ppm. Originally, the MASCOT server was used against the NCBInr for peptide mass fingerprinting (PMF). The criteria used to accept protein identifications were based on PMF data, namely the extent of sequence coverage, number of peptides matched, and score of probability. Protein identification was assigned when the following criteria were met: presence of at least four matching peptides and sequence coverage was greater than 15%.

**Table 1:** Proteins with increased expression levels in the SS biofilm, identified by MALDI-TOF/TOF MS.

Spot no.	Protein identified^a^	BLASTX similarity matched protein/species/identity score	Theoretical MW*/*pI^b^	Experimental MW*/*pI	Mascot score^c^	No. of Peptides matched^d^	Coverage (%)^e^	Fold change^f^		
								Mean	SD	*P* value
BF4	gi|146317813	Glyceraldehyde-3-phosphate dehydrogenase	35648/5.37	35000/5.40	280	24	62	2.5033	0.1955	0.006
BF5	gi|146317813	Glyceraldehyde-3-phosphate dehydrogenase	35648/5.37	35000/5.45	279	24	67	2.2967	0.1595	0.005
BF6	gi|253752311	UDP-N-acetylglucosamine 1-carboxyvinyltransferase 2	44720/5.28	41000/5.30	87	13	34	2.0667	0.0929	0.003
BF7	gi|253752506	Putative pyruvate dehydrogenase E1 component, alpha subunit	35240/5.25	36000/5.15	166	15	51	2.0833	0.1518	0.006
BF8	gi|146318280	Ornithine carbamoyltransferase	37832/5.26	40000/5.15	183	18	49	2.5067	0.1665	0.004
BF10	gi|146318058	Hypothetical protein SSU05_0403	31597/5.49	38000/5.00	304	21	86	2.2067	0.0611	0.001
BF14	gi|146319463	Enoyl-CoA hydratase	28643/5.31	25000/5.30	179	16	55	2.6367	0.3415	0.014

a) gi number in NCBI.

b) Theoretical pI was calculated using AnTheProt (http://antheprot-pbil.ibcp.fr/).

c) Mascot score obtained for the peptide mass fingerprint (PMF). The significance threshold was 70.

d) Number of peptides that match the predicted protein sequence.

e) Percentage of predicted protein sequence covered by matched peptides.

f) Differential protein expression (fold change) of corresponding protein between *Streptococcus suis* planktonic and bioflm proteome.

### Reverse Transcription (RT)-PCR

Total RNA was isolated from SS grown as biofilms and planktonic cells for 24 h with an E.Z.N.A.^TM^ bacterial RNA isolating kit (Omega, Beijing, China) following manufacturer’s directions. The RNA was subjected to DNase I (Promega, Madison, USA) treatment to remove DNA contaminantion. The cDNA synthesis was performed using the PrimeScript^TM^ RT reagent kit (TaKaRa, Shanghai, China) following manufacturer’s directions. mRNA levels were measured using two-step relative qRT-PCR. Relative mRNA amounts and expression ratios of selected genes were normalized to the expression of 16S rRNA mRNA amounts and fold changes were calculated as described by Gavrilin *et al.* [17]. A specific primer set was used to analyze GAPDH (F; 5′-CTTGGTAATCCCAGAATTGAACGG-3′ and R; 5′- TCATAGCAGCGTTTACTTCTTCAGC-3′), MRP (F; 5′- CAAGGAAAGTGAACAGAACGAGC-3′ and R; 5′- TAGTCGTCCAAACCTGAGTAGCG-3′) and 16S rRNA (F; 5′-GTTGCGAACGGGTGAGTAA-3′ and R; 5′-TCTCAGGTCGGCTATGTATCG-3′) mRNA content using the the SYBR Premix Ex Taq^TM^ Kit (Takara, Shanghai, China) following manufacturer’s instructions. Reactions were carried out in triplicate. An ABI 7300 RT-PCR system was used for relative qRT-PCR.

## Results

### Comparative Proteomics

**Figure 1:**
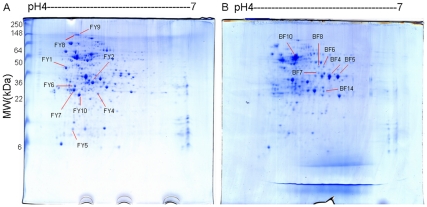
2D gel electrophoresis patterns of *Streptococcus suis* (SS) from whole cell lysate proteins. SS was grown as biofilms and planktonic conditions and the proteins separated by 2DGE. The proteins were separated in the first dimension by IEF (pH range 4-7) and in the second dimension by SDS-polyacrylamide gel electrophoresis. Molecular weight markers are on the left lane (kDa). (A) Protein pattern in the planktonic culture. (B) Protein pattern in the biofilm culture. Red arrow heads represent protein spots with a significantly (P < 0.05) increased amount in each culture mode.

2DGE of proteins from SS grown as biofilms or planktonic cells was performed to characterize the differences in protein expression between the two groups. The representative 2DGE images of biofilm and planktonic cells are provided in Figure 1. The majority of proteins were distributed in the range of pI 4–7 (Figures 1A and B). A total of 15 dominant protein spots were different between SS grown as biofilms or planktonic cells. MALDI-TOF-MS or MALDI-TOF/TOF-MS analysis identified 15 protein spots corresponding to 13 individual proteins. The probability score for the match, molecular weight (MW), isoelectric point (pI), number of peptide matches and the percentage of the total translated ORF sequence covered by the peptides were used as confidence factors in protein identification.

The proteins that were upregulated by more than two-fold included glyceraldehyde-3-phosphate dehydrogenase (GAPDH), UDP-N-acetylglucosamine 1-carboxyvinyltransferase 2 (MurA), pyruvate dehydrogenase E1 component (PDH), ornithine carbamoyltransferase (OTC), hypothetical protein SSU05_0403 and enoyl-CoA hydratase (Table 1). The proteins that were downregulated included ABC transporter periplasmic-binding protein (MntC), fructose-bisphosphate aldolase (FBA), dpr, BAA, muramidase-released protein (MRP), triosephosphate isomerase and elongation factor Tu (ET-Tu) (Table 2).

**Table 2:** Proteins with decreased expression levels in the SS biofilm, identified by MALDI-TOF/TOF MS.

Spot no.	Protein identified^a^	BLASTX similarity matched protein/species/identity score	Theoretical MW*/*pI^b^	Experimental MW*/*pI	Mascot score^c^	No. of Peptides matched^d^	Coverage (%)^e^	Fold change^f^		
								Mean	SD	*P* value
FY1	gi|146320941	ABC transporter periplasmicc-binding protein	36322/4.76	38500/4.60	208	18	58	3.8467	0.3384	0.005
FY2	gi|146320177	Fructose-bisphosphate aldolase	31136/4.90	33000/4.85	201	16	49	2.6467	0.1823	0.004
FY4	gi|27651368	Dpr	19583/4.91	22000/4.90	193	5	32	2.1667	0.2205	0.012
FY5	gi|1218040	BAA	16523/6.98	145000/4.75	123	8	42	2.2366	0.1060	0.002
FY6	gi|189037416	Elongation factor Tu	44727/4.87	39000/4.80	119	10	24	2.1733	0.2082	0.010
FY7	gi|146318185	Triosephosphate isomerase	26907/4.68	26000/4.70	91	11	36	2.5733	0.2059	0.006
FY8	gi|225625045	Muramidase-released protein	135693 /4.87	140000/4.80	158	9	52	2.4333	0.0950	0.001
FY9	gi|225625045	Muramidase-released protein	135693 /4.87	140000/4.85	158	9	52	2.3800	0.1769	0.005
FY10	gi|146318184	Elongation factor Tu	44727/4.87	34000/4.85	174	11	33	3.4200	0.2563	0.004

a) gi number in NCBI.

b) Theoretical pI was calculated using AnTheProt (http://antheprot-pbil.ibcp.fr/).

c) Mascot score obtained for the peptide mass fingerprint (PMF). The significance threshold was 70.

d) Number of peptides that match the predicted protein sequence.

e) Percentage of predicted protein sequence covered by matched peptides.

f) Differential protein expression (fold change) of corresponding protein between *Streptococcus suis* planktonic and bioflm proteome.

### Immunoreactive Proteins

**Figure 2:**
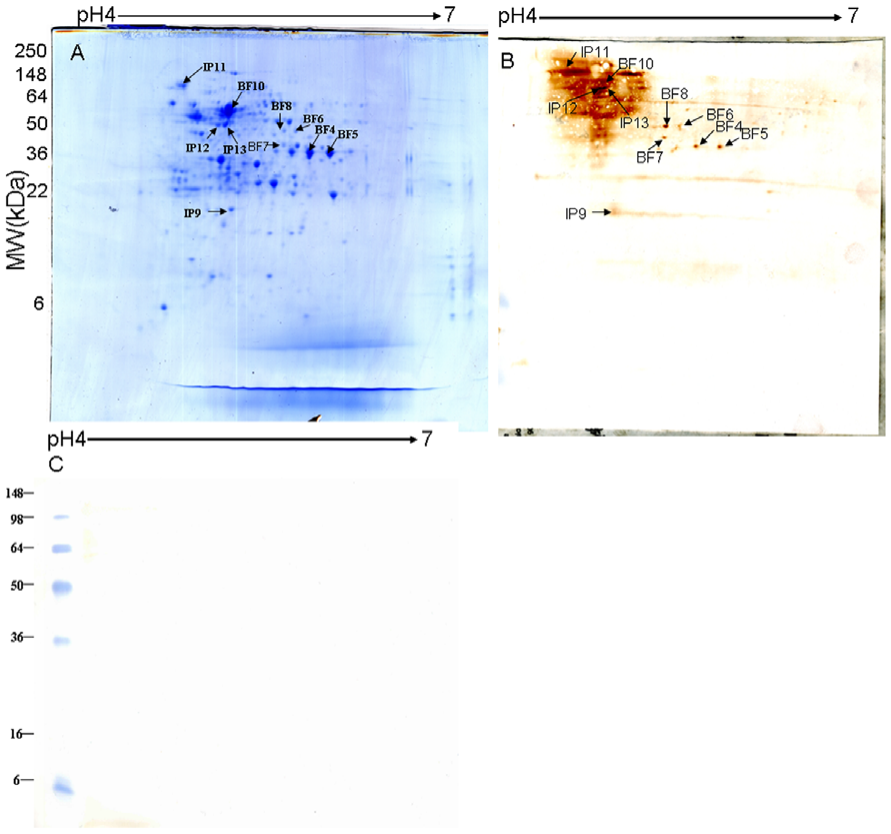
Gel electrophoresis of *Streptococcus suis* (SS) grown as biofilm cells with the immunoreactive proteins indicated. Preparative 2D gel of proteins from SS grown as biofilms and stained with CBB (A) or with western blot using convalescent serum (B) or preimmune sera (C). The identified proteins are indicated by pot number in Fig. 2A and B and Table 3. Molecular weight markers are on the left in kDa.

Ten immunoreactive protein spots were observed on the immunoblot of SS biofilm whole-cell proteins (Figure 2B) that matched the protein spots observed in the 2DGE gel (Figure 2A). When the blot was probed with preimmune sera, no specific immunoreactive protein spots were observed (Figure 2C). A total of 10 immunoreactive protein spots, corresponding to nine unique proteins, namely GAPDH, MurA, PDH, OTC, manganese-dependent superoxide dismutase (SodA), hypothetical protein SSU05_0403, molecular chaperone DnaK, hemolysin and phosphoglycerate kinase were identified (Table 3). Of these nine proteins, five (SodA, MurA, OTC, SSU05_0403, and phosphoglycerate kinase) have not been previously reported as immunoreactive proteins in SS to our knowledge. The remaining four immunogenic proteins (hemolysin, GAPDH, PDH and DnaK) have been identified in both planktonic and biofilm growth conditions in previous reports [12], [13], [14], [15].

**Table 3:** Immunoproteins identified by MALDI-TOF/TOF MS.

Spot no.	Protein identified^a^	BLASTX similarity matched protein/species/identity score	Theoretical MW*/*pI^b^	Experimental MW*/*pI	Mascot score^c^	No. of Peptides matched^d^	Coverage (%)^e^
BF4	gi|146317813	Glyceraldehyde-3-phosphate dehydrogenase	35648/5.37	35000/5.40	280	24	62
BF5	gi|146317813	Glyceraldehyde-3-phosphate dehydrogenase	35648/5.37	35000/5.45	280	24	67
BF6	gi|253752311	UDP-N-acetylglucosamine 1-carboxyvinyltransferase 2	44720/5.28	43000/5.30	202	13	34
BF7	gi|253752506	Putative pyruvate dehydrogenase E1 component, alpha subunit	35240/5.25	36000/5.15	166	15	51
BF8	gi|146318280	Ornithine carbamoyltransferase	37832/5.26	41000/5.15	183	18	49
IP9	gi|146319193	Manganese-dependent superoxide dismutase	21117/5.08	20000/4.90	101	9	68
BF10	gi|146318058	Hypothetical protein SSU05_0403	31597/5.49	48000/5.00	304	21	86
IP11	gi|146317956	Molecular chaperone DnaK	64787/4.62	64000/4.50	200	22	49
IP12	gi|146319057	Hemolysin	54 803/4.98	49000/4.85	109	10	27
IP13	gi|146317815	phosphoglycerate kinase	42048/4.85	47000/4.90	116	14	46

a) gi number in NCBI.

b) Theoretical pI was calculated using AnTheProt (http://antheprot-pbil.ibcp.fr/).

c) Mascot score obtained for the peptide mass fingerprint (PMF). The significance threshold was 70.

d) Number of peptides that match the predicted protein sequence.

e) Percentage of predicted protein sequence covered by matched peptides.

### Confirmation of Comparative Proteomics Results by Quantitative Real-time PCR

**Figure 3:**
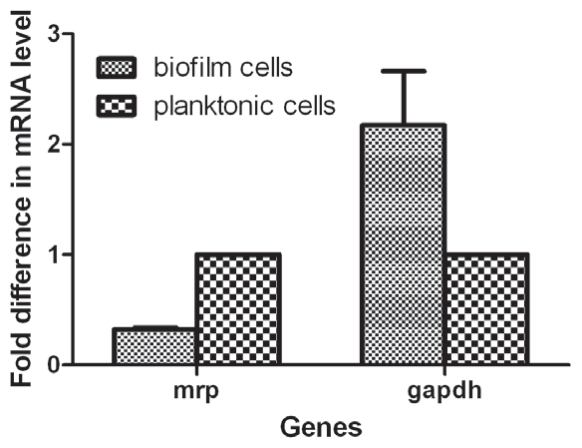
Glyceraldehyde-3-phosphate dehydrogenase (GAPDH) and Muramidase-released protein (MRP) mRNA amounts in *Streptococcus suis* grown as biofilms and planktonic cells. The mRNA content was analyzed by RT-PCR after adjusting for 16S rRNA mRNA content. The comparative cycle threshold method (2^−ΔΔCT^ method) was used to analyze the mRNA levels. Results are shown as fold changes compared to expression in the planktonic cell. Datas are the mean ± SEM for the results of three independent analysis.

Quantitative real-time PCR was performed on two selected genes to confirm the results of comparative proteomics analysis. We selected one upregulated gene (GAPDH) and one downregulated gene (MRP) in SS grown as biofilms. The qRT-PCR results confirmed the results of comparative proteomic analysis. SS grown as biofilms had 2.2 times higher GAPDH mRNA (*P* < 0.01) and 0.3 times lower MRP mRNA amounts (*P* < 0.05) than SS grown as planktonic cells (Figure 3).

## Discussion

In this study, the differences in the whole-cell protein expressions of SS grown under either biofilm or planktonic conditions were analyzed to reveal several differences in protein expressions between the two groups. Thirteen proteins, which showed differential expression under conditions of biofilm growth, were identified. Of the 13 proteins, six proteins were up-regulated and seven proteins were down-regulated in the biofilm proteome. Similar results have been demonstrated using other bacteria [18], [19], [20], [21]. For example, nine proteins are up-regulated in the *streptococcus mutans* biofilm cells compared with the planktonic cells [16]. Similarly, Alen *et al.* reported that eight proteins are up-regulated and four proteins are down-regulated in the *Neisseria meningitidis* biofilm [22]. In this study, though some other proteins were either down-regulated or up-regulated between the two groups, we only chose the 13 proteins because these 13 proteins were consistently different between triplicate gels. Using proteins from the biofilm cells and immunoblotting with convalescent sera, nine immunogenic proteins were identified. Only a limited number of proteins were identified, which may be due to serum being collected at early stages of infection in this study. Serum collected at late stages of infections identifies more protein spots [23].

Although bacteria in biofilms exhibit persistence in spite of sustained host defenses, little is known about the host immune response to biofilm infections. Protein expression in biofilms grown *in vivo* cannot be easily studied because it is difficult to extract bacterial proteins from *in vivo* grown biofilms. Certain antibodies may prevent biofilm development. For example, an antibody to an outer membrane protein in *Pseudomonas aeruginosa* was recently shown to inhibit biofilm formation by interfering with adhesion to the surface [23]. We employed a system in which mini-pigs were challenged with SS. By collecting sera from these mini-pigs during the course of infection and utilizing these sera to probe immunoblots of protein isolated from the *in vitro*-grown biofilm, we were able to visualize those immunogenic proteins that were present during biofilm infection. Though there are studies describing the immunogens present on the surface of planktonic SS, the data presented in this paper are the first to describe biofilm-specific proteins recognized by host antibodies. We found 10 immunoreactive spots that corresponded to nine individual immunogenic proteins. It was very interesting that five identified immunogenic proteins were up-regulated in the *Streptococcus* biofilm. A similar result has been found in *S. aureus*, where approximately 76% of the immunogenic proteins were upregulated in at least one of the stages of biofilm formation during *in vitro* growth [23]. Previous studies have evaluated the immunogenicity of SS proteins in planktonic growth conditions [13], [14], [15]. However, these studies failed to detect the biofilm-associated antigens found in this work, with the exception of hemolysin, GAPDH, PDH and DnaK. The above four common immunogenic proteins were identified in both growth conditions and hence could be promising vaccine candidates to prevent both biofilm infections and acute infections. The remaining immunoreactive proteins in SS2 found in this study have not been previously reported to our knowledge.

Future studies should focus on identifying the role of GAPDH, MntC, OTC, FBA and PDH in biofilm formation, because Puttamreddy reported that biofilm formation and cellular adherence to epithelial cells are interlinked [24]. A previous study showed that these proteins could mediate cell adherence. GAPDH and MntC mutant strains confirmed the speculation [25]. Therefore, it is reasonable to think that other proteins might be involved in biofilm formation of SS. Study of the other proteins is ongoing in our laboratory to check if they are related to biofilm formation.

GAPDH is a glycolytic enzyme responsible for the conversion of glyceraldehyde 3-phosphate into 1,3-diphosphoglycerate. GAPDH is a SS surface protein and mediates cell adhesion and plays an important role in bacterial infection and invasion [26], [27]. GADPH was upregulated in SS grown as biofilms. Similarly, biofilms of *Pseudomonas aeruginosa* [28] and *Staphylococus xylosus* [29] upregulate GAPDH. This also resembles the regulation of the enzyme in *E. coli* K12 under microaerobic conditions [30], which is indirectly linked to oxygen limitation in biofilms. Furthermore, SS mutants with GAPDH knocked-out had decreased ability to form biofilms (data not shown). It has also been reported that GADPH is an immunogenic protein found on the cell wall of SS [15]. GAPDH is reported in the development of subunit vaccines against *Edwardsiella tarda* [31], [32], *Streptococcus pneumoniae* [33] and *Bacillus anthracis* [34].

The protein from spot BF8 matched SS OTC. OTC is a key enzyme in the urea cycle and detoxifies ammonium produced from amino acid catabolism [35], [36]. In *Bacillus cereus,* OTC was upregulated in biofilm cells at 18 h of culture. This may be indicative of oxygen depletion in microcolonies, or alternatively, it may indicate that the attached cells were preparing for growth within a biofilm before the conditions became anoxic. OTC is a putative adhesin for *Staphylococcus epidermidis* [37] and has been identified as an immunogenic protein from the outer surface protein preparations of *S. agalactiae*, *S. pyogenes* and *Clostridium perfringens* [38].

MntC is part of the MntABC transporter and is involved in oxidative stress defense in *Nisseria gonorrhoeae* and *Nisseria meningitidis* [39]. The *N. gonorrhoeae* MntC knock-out is more sensitive to oxygen killing, and accumulate less manganese than the wild type [39]. Furthermore, the gonococcal MntC knock-out have reduced intracellular survival and have reduced ability to form biofilms [25]. MntC facilitates biofilm formation of Gonococci, and affects the colonization of mice [40]. Alen *et al.* reported that biofilm formation is almost completely abrogated in the MntC mutants of *Neisseria meningitides* [22].

PDH converts pyruvate to acetyl coenzyme A, which is subsequently used in the tricarboxylic acid cycle to generate NADH, ATP, and reduced flavin adenine dinucleotide [41]. Welin et al. [42] and Korithoski et al. [43] used 2DGE to reveal that PDH is upregulated 2.5-fold in *S.mutans* biofilm cells. PDH is thought to play a role in the binding to fibronectin [44]. PDH is an important part of the cytoskeleton of *M. pneumoniae* and is linked to cell adhesion [45]. PDH is highly immunogenic in other bacterial species, such as *N. meningitidis* [46], *Mycoplasma capricolum* [47] and *M. hyopneumoniae* [48]. Recently, PDH has been tested as a DNA vaccine against *M. mycoides* subsp. mycoides, the causal agent of contagious bovine pleuropneumonia [49].

The upregulation of SodA involved in detoxification of ROS was in line with proteomic and microarray studies in biofilms of other bacteria; *e.g., Staphylococcus aureus* and *Neisseria meningitidis* [10], [22]. SodA has a role in the protection of group A streptococcus challenge [50]. A similar result was shown with *Listeria monocytogenes* [51], *Brucella abortus* [52] and *Escherichia coli* [53]. Recombinant SodA elicits strong antibody responses in mice [53].

MurA is a key enzyme involved in bacterial cell wall peptidoglycan synthesis and a target for the antimicrobial agent, fosfomycin. Increased expression of MurA in the biofilms may contribute to the increased drug resistance [54].

The BLASTx search identified IP11 as molecular chaperone DnaK, IP12 as hemolysin and BF13 as phosphoglycerate kinase. DnaK is an important immunogen in *S. pneumoniae* [55] and *S. pyogenes* [56]. Hemolysin is a secreted protein and is a bacterial virulence factor [57]. Phosphoglycerate kinase is a major outer surface protein of *S. suis.* The above three proteins have been reported to be immunogenic in SS [13].

In this study, most of the downregulated genes such as FY1, FY2, FY4, FY5, FY6, FY7, and FY8 are likely to be involved in protein synthesis or encode membrane proteins/transporters (Table 2). This reduced level of expression may indicate a limited bacterial growth rate and that the SS organisms in biofilm environments have limited but more specific metabolic activity. Among the down-regulated genes was FY2 which represents fructose-bisphosphate aldolase. Fructoses are extracellular storage compounds and can act as binding sites for bacterial adhesion [58], [59]. Extracellular fructans play a role in sucrose-dependent bacterial adherence and biofilm accumulation. To down-regulate this sucrose-dependent cell–cell adhesion, biofilm formation gene in biofilm cells makes bio-economic sense since sucrose is absent in the environment. FBA and MRP are virulence factors in a variety of organisms [60]. The expression of virulence factors in the planktonic cells will make the planktonic cells more virulent and, therefore, cause acute infections than biofilm cells [61]. In our previous study, biofilm cells had lower virulence when compared to planktonic cells in an animal model. In addition some virulence genes were downregulated in biofilm cells [8]. Changes in the structure of the bacteria may alter the expression levels of virulence genes. Biofilm cells are wrapped by a polysaccharide complex, which would influence the virulence factors secreted from the bacteria.

## References

[1] Gottschalk M, Xu J, Calzas C, Segura M (2010). Streptococcus suis: a new emerging or an old neglected zoonotic pathogen?. Future Microbiol.

[2] Staats JJ, Feder I, Okwumabua O, Chengappa MM (1997). Streptococcus suis: past and present.. Vet Res Commun.

[3] Esgleas M, Lacouture S, Gottschalk M (2005). Streptococcus suis serotype 2 binding to extracellular matrix proteins.. FEMS Microbiol Lett.

[4] Charland N, Nizet V, Rubens CE, Kim KS, Lacouture S (2000). Streptococcus suis serotype 2 interactions with human brain microvascular endothelial cells.. Infect Immun.

[5] Benga L, Goethe R, Rohde M, Valentin-Weigand P (2004). Non-encapsulated strains reveal novel insights in invasion and survival of Streptococcus suis in epithelial cells.. Cell Microbiol.

[6] Brown MR, Allison DG, Gilbert P (1988). Resistance of bacterial biofilms to antibiotics: a growth-rate related effect?. J Antimicrob Chemother.

[7] Brady RA, Leid JG, Calhoun JH, Costerton JW, Shirtliff ME (2008). Osteomyelitis and the role of biofilms in chronic infection.. FEMS Immunol Med Microbiol.

[8] Wang Y, Zhang W, Wu Z, Lu C (2011). Reduced virulence is an important characteristic of biofilm infection of Streptococcus suis.. FEMS Microbiol Lett.

[9] Parsek MR, Singh PK (2003). Bacterial biofilms: an emerging link to disease pathogenesis.. Annu Rev Microbiol.

[10] Resch A, Rosenstein R, Nerz C, Gotz F (2005). Differential gene expression profiling of Staphylococcus aureus cultivated under biofilm and planktonic conditions.. Appl Environ Microbiol.

[11] Uppuluri P, Chaturvedi AK, Srinivasan A, Banerjee M, Ramasubramaniam AK (2010). Dispersion as an important step in the Candida albicans biofilm developmental cycle.. PLoS Pathog.

[12] Zhang W, Lu CP (2007). Immunoproteomic assay of membrane-associated proteins of Streptococcus suis type 2 China vaccine strain HA9801.. Zoonoses Public Health.

[13] Zhang W, Lu CP (2007). Immunoproteomics of extracellular proteins of Chinese virulent strains of Streptococcus suis type 2.. Proteomics.

[14] Geng H, Zhu L, Yuan Y, Zhang W, Li W (2008). Identification and characterization of novel immunogenic proteins of Streptococcus suis serotype 2.. J Proteome Res.

[15] Zhang A, Xie C, Chen H, Jin M (2008). Identification of immunogenic cell wall-associated proteins of Streptococcus suis serotype 2.. Proteomics.

[16] Rathsam C, Eaton RE, Simpson CL, Browne GV, Valova VA (2005). Two-dimensional fluorescence difference gel electrophoretic analysis of Streptococcus mutans biofilms.. J Proteome Res.

[17] Gavrilin MA, Deucher MF, Boeckman F, Kolattukudy PE (2000). Monocyte chemotactic protein 1 upregulates IL-1beta expression in human monocytes.. Biochem Biophys Res Commun.

[18] Shin JH, Lee HW, Kim SM, Kim J (2009). Proteomic analysis of Acinetobacter baumannii in biofilm and planktonic growth mode.. J Microbiol.

[19] Oosthuizen MC, Steyn B, Theron J, Cosette P, Lindsay D (2002). Proteomic analysis reveals differential protein expression by Bacillus cereus during biofilm formation.. Appl Environ Microbiol.

[20] Kalmokoff M, Lanthier P, Tremblay TL, Foss M, Lau PC (2006). Proteomic analysis of Campylobacter jejuni 11168 biofilms reveals a role for the motility complex in biofilm formation.. J Bacteriol.

[21] Svensater G, Welin J, Wilkins JC, Beighton D, Hamilton IR (2001). Protein expression by planktonic and biofilm cells of Streptococcus mutans.. FEMS Microbiol Lett.

[22] van Alen T, Claus H, Zahedi RP, Groh J, Blazyca H (2010). Comparative proteomic analysis of biofilm and planktonic cells of Neisseria meningitidis.. Proteomics.

[23] Brady RA, Leid JG, Camper AK, Costerton JW, Shirtliff ME (2006). Identification of Staphylococcus aureus proteins recognized by the antibody-mediated immune response to a biofilm infection.. Infect Immun.

[24] Puttamreddy S, Cornick NA, Minion FC (2010). Genome-wide transposon mutagenesis reveals a role for pO157 genes in biofilm development in Escherichia coli O157:H7 EDL933.. Infect Immun.

[25] Lim KH, Jones CE, vanden Hoven RN, Edwards JL, Falsetta ML (2008). Metal binding specificity of the MntABC permease of Neisseria gonorrhoeae and its influence on bacterial growth and interaction with cervical epithelial cells.. Infect Immun.

[26] Brassard J, Gottschalk M, Quessy S (2004). Cloning and purification of the Streptococcus suis serotype 2 glyceraldehyde-3-phosphate dehydrogenase and its involvement as an adhesin.. Vet Microbiol.

[27] Wang K, Lu C (2007). Adhesion activity of glyceraldehyde-3-phosphate dehydrogenase in a Chinese Streptococcus suis type 2 strain.. Berl Munch Tierarztl Wochenschr.

[28] Sauer K, Camper AK, Ehrlich GD, Costerton JW, Davies DG (2002). Pseudomonas aeruginosa displays multiple phenotypes during development as a biofilm.. J Bacteriol.

[29] Planchon S, Desvaux M, Chafsey I, Chambon C, Leroy S (2009). Comparative subproteome analyses of planktonic and sessile Staphylococcus xylosus C2a: new insight in cell physiology of a coagulase-negative Staphylococcus in biofilm.. J Proteome Res.

[30] Peng L, Shimizu K (2003). Global metabolic regulation analysis for Escherichia coli K12 based on protein expression by 2-dimensional electrophoresis and enzyme activity measurement.. Appl Microbiol Biotechnol.

[31] Kawai K, Liu Y, Ohnishi K, Oshima S (2004). A conserved 37 kDa outer membrane protein of Edwardsiella tarda is an effective vaccine candidate.. Vaccine.

[32] Liu Y, Oshima S, Kurohara K, Ohnishi K, Kawai K (2005). Vaccine efficacy of recombinant GAPDH of Edwardsiella tarda against Edwardsiellosis.. Microbiol Immunol.

[33] Jomaa M, Kyd JM, Cripps AW (2005). Mucosal immunisation with novel Streptococcus pneumoniae protein antigens enhances bacterial clearance in an acute mouse lung infection model.. FEMS Immunol Med Microbiol.

[34] Delvecchio VG, Connolly JP, Alefantis TG, Walz A, Quan MA (2006). Proteomic profiling and identification of immunodominant spore antigens of Bacillus anthracis, Bacillus cereus, and Bacillus thuringiensis.. Appl Environ Microbiol.

[35] Yu W, Lin Y, Yao J, Huang W, Lei Q (2009). Lysine 88 acetylation negatively regulates ornithine carbamoyltransferase activity in response to nutrient signals.. J Biol Chem.

[36] Yu HJ, Liu XC, Wang SW, Liu LG, Zu RQ (2005). [Matched case-control study for risk factors of human Streptococcus suis infection in Sichuan Province, China].. Zhonghua Liu Xing Bing Xue Za Zhi.

[37] Hussain M, Peters G, Chhatwal GS, Herrmann M (1999). A lithium chloride-extracted, broad-spectrum-adhesive 42-kilodalton protein of Staphylococcus epidermidis is ornithine carbamoyltransferase.. Infect Immun.

[38] Alam SI, Bansod S, Kumar RB, Sengupta N, Singh L (2009). Differential proteomic analysis of Clostridium perfringens ATCC13124; identification of dominant, surface and structure associated proteins.. BMC Microbiol.

[39] Tseng HJ, Srikhanta Y, McEwan AG, Jennings MP (2001). Accumulation of manganese in Neisseria gonorrhoeae correlates with resistance to oxidative killing by superoxide anion and is independent of superoxide dismutase activity.. Mol Microbiol.

[40] Wu H, Soler-Garcia AA, Jerse AE (2009). A strain-specific catalase mutation and mutation of the metal-binding transporter gene mntC attenuate Neisseria gonorrhoeae in vivo but not by increasing susceptibility to oxidative killing by phagocytes.. Infect Immun.

[41] Domingo GJ, Chauhan HJ, Lessard IA, Fuller C, Perham RN (1999). Self-assembly and catalytic activity of the pyruvate dehydrogenase multienzyme complex from Bacillus stearothermophilus.. Eur J Biochem.

[42] Welin J, Wilkins JC, Beighton D, Wrzesinski K, Fey SJ (2003). Effect of acid shock on protein expression by biofilm cells of Streptococcus mutans.. FEMS Microbiol Lett.

[43] Korithoski B, Levesque CM, Cvitkovitch DG (2008). The involvement of the pyruvate dehydrogenase E1alpha subunit, in Streptococcus mutans acid tolerance.. FEMS Microbiol Lett.

[44] Savini V, Catavitello C, Astolfi D, Balbinot A, Masciarelli G (2010). Bacterial contamination of platelets and septic transfusions: review of the literature and discussion on recent patents about biofilm treatment.. Recent Pat Antiinfect Drug Discov.

[45] Francolini I, Donelli G (2010). Prevention and control of biofilm-based medical-device-related infections.. FEMS Immunol Med Microbiol.

[46] Sen A, Hu C, Urbach E, Wang-Buhler J, Yang Y (2001). Cloning, sequencing, and characterization of CYP1A1 cDNA from leaping mullet (Liza Saliens) liver and implications for the potential functions of its conserved amino acids.. J Biochem Mol Toxicol.

[47] Zhu PP, Peterkofsky A (1996). Sequence and organization of genes encoding enzymes involved in pyruvate metabolism in Mycoplasma capricolum.. Protein Sci.

[48] Matic JN, Wilton JL, Towers RJ, Scarman AL, Minion FC (2003). The pyruvate dehydrogenase complex of Mycoplasma hyopneumoniae contains a novel lipoyl domain arrangement.. Gene.

[49] March JB, Jepson CD, Clark JR, Totsika M, Calcutt MJ (2006). Phage library screening for the rapid identification and in vivo testing of candidate genes for a DNA vaccine against Mycoplasma mycoides subsp. mycoides small colony biotype.. Infect Immun.

[50] McMillan DJ, Davies MR, Good MF, Sriprakash KS (2004). Immune response to superoxide dismutase in group A streptococcal infection.. FEMS Immunol Med Microbiol.

[51] Hess J, Dietrich G, Gentschev I, Miko D, Goebel W (1997). Protection against murine listeriosis by an attenuated recombinant Salmonella typhimurium vaccine strain that secretes the naturally somatic antigen superoxide dismutase.. Infect Immun.

[52] Vemulapalli R, He Y, Boyle SM, Sriranganathan N, Schurig GG (2000). Brucella abortus strain RB51 as a vector for heterologous protein expression and induction of specific Th1 type immune responses.. Infect Immun.

[53] Onate AA, Vemulapalli R, Andrews E, Schurig GG, Boyle S (1999). Vaccination with live Escherichia coli expressing Brucella abortus Cu/Zn superoxide dismutase protects mice against virulent B. abortus.. Infect Immun.

[54] Kumar S, Parvathi A, Hernandez RL, Cadle KM, Varela MF (2009). Identification of a novel UDP-N-acetylglucosamine enolpyruvyl transferase (MurA) from Vibrio fischeri that confers high fosfomycin resistance in Escherichia coli.. Arch Microbiol.

[55] Kim SW, Choi IH, Kim SN, Kim YH, Pyo SN (1998). Molecular cloning, expression, and characterization of dnaK in Streptococcus pneumoniae.. FEMS Microbiol Lett.

[56] Lemos JA, Burne RA, Castro AC (2000). Molecular cloning, purification and immunological responses of recombinants GroEL and DnaK from Streptococcus pyogenes.. FEMS Immunol Med Microbiol.

[57] Pernot L, Chesnel L, Le Gouellec A, Croize J, Vernet T (2004). A PBP2x from a clinical isolate of Streptococcus pneumoniae exhibits an alternative mechanism for reduction of susceptibility to beta-lactam antibiotics.. J Biol Chem.

[58] Blom NS, Tetreault S, Coulombe R, Sygusch J (1996). Novel active site in Escherichia coli fructose 1,6-bisphosphate aldolase.. Nat Struct Biol.

[59] Rozen R, Bachrach G, Bronshteyn M, Gedalia I, Steinberg D (2001). The role of fructans on dental biofilm formation by Streptococcus sobrinus, Streptococcus mutans, Streptococcus gordonii and Actinomyces viscosus.. FEMS Microbiol Lett.

[60] Pancholi V, Chhatwal GS (2003). Housekeeping enzymes as virulence factors for pathogens.. Int J Med Microbiol.

[61] Wijeratne AJ, Zhang W, Sun Y, Liu W, Albert R (2007). Differential gene expression in Arabidopsis wild-type and mutant anthers: insights into anther cell differentiation and regulatory networks.. Plant J.

